# Household Polluting Fuel Use and Frailty among Older Adults in Rural China: The Moderating Role of Healthy Lifestyle Behaviors

**DOI:** 10.3390/healthcare11121747

**Published:** 2023-06-14

**Authors:** Huiying Chen, Xinpeng Xu, Cangcang Jia, Hai Gu, Lu Zhang, Yang Yi

**Affiliations:** 1 Research Center for Health Policy and Management, Nanjing University, Nanjing 210093, China; 2 School of Public Health, Nanjing Medical University, Nanjing 211166, China; 3 School of Health Policy and Management, Nanjing Medical University, Nanjing 211166, China

**Keywords:** older adults, frailty index, household air pollution, polluting cooking fuels, healthy lifestyle behaviors

## Abstract

This study worked to investigate the effect of household polluting fuel use (HPFU), as an indicator of household air pollution exposure, on frailty among older adults in rural China. Additionally, this study aimed to examine the moderating effect of healthy lifestyle behaviors on the aforementioned association. This study employed cross-sectional data from the 2018 Chinese Longitudinal Healthy Longevity Survey, which conducted nationally representative sampling of older adults from 23 provinces in mainland China. The frailty index was calculated using 38 baseline variables that assessed health deficits through questionnaire surveys and health examinations. A total of 4535 older adults aged 65 years and above were included in our study, among whom, 1780 reported using polluting fuels as their primary household cooking fuel. The results of regression analyses and multiple robustness checks indicated a significant increase in the frailty index due to HPFU. This environmental health threat was more profound among women, illiterate individuals, and low-economic-status groups. Moreover, healthy dietary and social activities had significant moderating effects on the association between HPFU and frailty. HPFU can be regarded as a risk factor for frailty among older adults in rural China, with its effects exhibiting socio-economic disparities. The adoption of healthy lifestyle behaviors can alleviate the frailty associated with HPFU. Our findings underscore the significance of using clean fuels and improving household air quality for healthy aging in rural China.

## 1. Introduction

As the process of aging accelerates, China has more than 190 million people aged 65 and older, accounting for an increase of 4.63 percentage points in the proportion of the total population over the past decade [1]. By 2030, it is anticipated that older adults in China will bear 65.6% of the total burden of disease, imposing significant pressure on the healthcare system [2]. Frailty is considered a common characteristic of the aging process. As individuals age, the physiological reserves of multiple systems decrease and may become dysregulated, impeding their ability to store and resist stressors. This can lead to adverse health outcomes, such as disability, dependence, falls, need for long-term care, and mortality [3]. Among the various clusters, the physical frailty phenotype has been extensively studied, encompassing weight loss, low grip strength, exhaustion, and slow motor performance [4]. The frailty index is also a commonly used assessment method. According to Rockwood, frailty can be defined in relation to the accumulation of deficits, primarily depending on the number of accumulated deficits rather than their nature [5]. In practice, most studies compute an individual’s frailty index as the proportion of cumulative health deficits to the total number of deficits. Given that frailty is a major focus of prevention, rehabilitation, and public health programs for older adults [6], identifying risk factors that influence frailty can aid in the prevention and management of frailty conditions.

Environmental exposures inherently drive the aging process. Air pollutants alter the relationship between successful aging and pathological aging by inducing biological pathways, such as inflammation and hormonal changes [7]. Short- or long-term exposure to inhalable particulate matter (PM) in the air may cause, or accelerate, age-related diseases [8,9]. Recent studies have shown that heavy metals in the environment, such as blood lead, may increase the risk of functional disability, leading to frailty in the oldest adults (age ≥ 80 years) [10]. Lee further demonstrated that frailty among other vulnerable groups, such as those with a higher burden of disease or those residing in suburban areas, is also related to air pollutants [11].

Research has indicated that rural residents are more susceptible to the effects of household air pollution (HAP) caused by the burning of polluting fuels. The aging process impairs both physical and social functions [12], which, when combined with prolonged exposure to HAP, renders older adults even more sensitive to household air quality problems. Several epidemiological studies have identified a correlation between polluting fuel use and physical function, including possible decreases in life expectancy [13] and increases in mortality rates [14]. However, the assessment of health changes caused by aging through a single disease model is insufficient. A practical multidimensional health indicator, such as the frailty index, may be more appropriate. Although there is limited quantitative research, a recent study confirmed that cooking with solid fuels may be a risk factor for phenotypic frailty, such as weakness, sluggishness, and weight loss [15].

Given that frailty is a dynamic process that can deteriorate or improve over time, research has demonstrated that its negative effects can be reversed, managed, and prevented [16]. Consequently, reducing factors that worsen frailty or enhancing those that alleviate frailty may be more essential, as it can help to identify appropriate intervention strategies. Some intervention studies have focused on testing hypotheses on modifiable behavioral factors in frailty transitions [17,18]. Among these is the adoption of healthy lifestyle behaviors, such as socialization and adequate nutrition. Traditional leisure activities, such as playing cards or mahjong, have been found to be effective interventions for alleviating loneliness among Chinese adults [19]. Furthermore, improving diet quality during middle age is associated with better physical functioning in later life [20]. Consequently, in rural areas with high levels of HAP, adopting healthy lifestyle behaviors might help prevent or delay age-related health problems.

To summarize, HAP caused by household polluting fuel use (HPFU) may pose a significant risk to the frailty of older adults, despite the limited evidence supporting this association. In this study, we employed nationwide representative data to achieve the following objectives. First, this study aims to investigate the potential association between HPFU and frailty among older adults in rural China. We utilized the frailty index, a widely recognized instrument consisting of 38 items covering a wide range of physical and mental aspects, to assess the level of frailty in older adults. Notably, we gave particular attention to psychological vulnerability among older adults by integrating the CES-D and GAD-7 scales into our evaluation. Second, to complement the evidence on socio-economic inequality associated with HAP-related frailty, we utilized heterogeneity analysis to identify vulnerable subpopulations. We considered that gender, educational level, and economic status may modify the effects of this association. Third, to investigate the moderating effect of healthy lifestyle behaviors, we analyzed the correlation between HPFU, healthy lifestyle behaviors, and the frailty index to enrich the relevant research.

## 2. Materials and Methods

### 2.1. Data Source and Sample Selection

The Chinese Longitudinal Healthy Longevity Survey (CLHLS) was launched in 1998 by the Center for Healthy Aging and Development Research at the National School of Development of Peking University, with follow-up surveys conducted at intervals of 2–3 years [21]. Unlike other official statistics published in China, CLHLS takes special care to ensure adequate representation of the oldest members of the population in its national survey. Individuals aged 80 years and above constituted 67.4% of the total sample, providing an excellent basis for assessing the health of Chinese elders. Given its longstanding history as the earliest social science survey to be held in mainland China, CLHLS is widely regarded as a vital resource for interdisciplinary research and policy analysis concerning healthy aging [22]. Our study is based on the latest 2018 CLHLS, which incorporated a series of questionnaire items gauging socio-economic background, health and medical care, lifestyle, and more sophisticated health examinations. Ideal for thorough analysis, we only selected rural residents aged 65 years and older who had not missed any key variables. The household registration, also known the as “hukou” system, serves as the prominent basis for Chinese residents obtaining fundamental healthcare services, labor and employment opportunities, basic public education, and other rights [23]. In our study, rural residents were defined as individuals whose hukou designates them as agricultural. They primarily reside and work in rural areas and frequently engage in agriculture, forestry, fishing, and other related activities as their primary source of income and livelihood. The total number of valid samples we collated was 4535, of which, 1780 used polluting fuels for household cooking while 2755 utilized clean fuels.

### 2.2. Measures

#### 2.2.1. Dependent Variable: Frailty Index

In order to comprehensively assess an individual’s cumulative deficits, various dimensions of health have been incorporated into the calculation of the frailty index [24]. Following established research [25,26], our investigation utilized 2018 CLHLS to compile a frailty index consisting of 38 items. These items included an individual’s self-rated health, interviewee-rated health, hearing and visual function, sleep quality, tooth situation, heart rhythm, waist-to-hip ratio, body-mass index, serious illnesses number, numerous chronic diseases, depression, and anxiety (please see Table A1 in Appendix A). As a systemic phenomenon, the construction of the frailty index cannot ignore the vital role of psychological and mental abilities in maintaining functionality [27]. Mcdougall highlighted the need to pay particular attention to the presence of depression and anxiety [28]. Hence, our study incorporated 7 items from the Center for Epidemiological Studies Depression Scale (CES-D), as well as another 7 items derived from the Generalized Anxiety Disorder Scale (GAD-7), rather than purely physical items. Our study differs from previous research in the number and types of indicators used, such as the exclusion of measures for social and oral frailty. However, Rockwood affirmed that the specific deficits chosen are not critical, since a random selection of variables will still yield comparable results.

In order to gauge the degree of frailty, every variable in our frailty index was categorized as either dichotomous or multichotomous and mapped to the 0.00–1.00 interval. Here, the designation of 0.00 denoted the healthiest state whereas 1.00 highlighted the ultimate level of frailty, that is, the presence of maximum deficits. In accordance with Goggins, participants with two or more serious illnesses were allotted a value of 2.00 [29]. In accordance with the consensus on constructing the frailty index, we did not assign weights to correlated variables since the specific defects themselves appear less important than the overall failure of the organism (i.e., the number of defects) [30]. Then, the frailty index was calculated by adding up all deficits and dividing the sum by the total number of deficits (n = 39). Consequently, the frailty index is a continuous variable, extending from 0 to 1. Participants were regarded as being in a more precarious and susceptible condition if their frailty index values were higher.

#### 2.2.2. Independent Variable: Household Polluting Fuel Use

The World Health Organization attributes HAP to the inefficient and polluting fuels used in and around homes, frequently comprising solid fuels, such as wood, coal, charcoal, crop waste, and dung, as well as kerosene [31]. Based on this definition, our research evaluated HPFU by inquiring about respondents’ typical fuel choices for domestic cooking with the question: “Which fuels are normally used for cooking in your home?”. For responses marked “Kerosene,” “Coal,” “Charcoal,” or “Firewood,” HPFU was assigned a value of 1; conversely, a value of 0 was assigned in all other cases. Users who declared “Never cooking” and those with incomplete questionnaire responses were excluded.

#### 2.2.3. Moderating Variable: Healthy Lifestyle Behaviors

The maintenance of a healthy lifestyle among older adults is a multifaceted undertaking that requires both a nutritious diet and active participation in social activities. In accordance with previous research [32], “Fresh vegetables” and “Fresh fruits” were treated as binary variables, with a value of 1 indicating daily consumption and 0 representing no intake. The term “Food variety” referred to the number of different types of food consumed by the respondent per day. The term “Balanced diet” was a binary variable, with a value of 1, indicating the simultaneous consumption of vegetables, fruits, meat, eggs, and fish on a daily basis; meanwhile, a value of 0 denoted the lack of such intake. Participation in social activities was assessed by evaluating whether respondents engaged in leisurely pursuits, such as gardening, reading, social interaction, or card-playing. These activities were transformed into binary variables based on participant responses, with 1 indicating yes and 0 indicating no.

#### 2.2.4. Control Variables

With reference to previous work, individual demographics, socioeconomic status, health risks, and residential environment could affect the frailty index in older adults [33]. Therefore, our study chose to incorporate control variables based on these four categories. The individual demographic characteristics comprised gender, age, ethnicity, and marital status. Marital status was specified as a binary variable where a value of 1 indicated that the respondent was married while a value of 0 indicated that the respondent was single, divorced, or widowed. Socioeconomic status was assessed based on education level, financial independence, and economic status. Education level was categorized into three dummy variables, namely, illiterate, possessing primary education, and having received at least a middle school education. Respondents were queried about their main source of financial support, which was subsequently recoded as a binary variable, with 1 representing retirement wages or labor earnings and 0 representing other sources. Similarly, respondents were asked to report their perceived level of affluence, categorized as 1 if they considered themselves relatively or very prosperous, and 0 if otherwise. Health risks comprised an activities of daily living (ADL) score, smoking, drinking, and exercise. For ADL tasks, such as bathing, dressing, toileting, indoor mobility, eating, and incontinence, a score of 2 represented total independence, 1 denoted partial assistance required, and 0 indicated complete dependence. The ADL score ranged from 0 to 12, with higher scores indicating greater independence for the respondents in performing ADL. Lastly, we introduced two dummy variables, ventilation and musty odor, to capture the living environment, with a value of 1 indicating yes and a value of 0 indicating no.

### 2.3. Statistical Analyses

#### 2.3.1. Descriptive Statistical Analysis and Difference Test

We used Stata software (version 14.0, StataCorp, College Station, TX, USA) for descriptive statistics to examine the basic characteristics of participation in rural China. Subsequently, we used the *t*-test to assess the difference in frailty index between those who used polluting fuels and those who did not use such fuels. Here, p≤0.1 indicated statistical significance. Consistent with previous studies [34,35], we used a slightly relaxed significance level. This allowed us to capture potentially important findings while avoiding the over-rejection of null hypotheses.

#### 2.3.2. Model Construction

Regression analysis was performed to investigate the influence of HPFU on the frailty index. The model is as follows:

(1) $${Frialty}_{i}=\alpha_{0}+\beta_{0}{HPFU}_{i}+\delta X_{i}^{\prime }+\varepsilon_{i}$$

among them, ${Frialty}_{i}$ represents an individual’s frailty index; ${HPFU}_{i}$ denotes the use of polluting fuels for household cooking; $X_{i}^{\prime }$ is a series of control variables affecting the frailty index, including individual demographics, socioeconomic status, health risks, and residential environment; $\varepsilon_{i}$ is the random disturbance term in the model; and $\beta_{0}$ stands for the regression coefficient that represents the effect of HPFU on the frailty index.

In addition, we employed a propensity score matching (PSM) method to overcome potential confounding variables related to both HPFU and the frailty index. Following the PSM analysis paradigm [36], we first calculated propensity scores utilizing logit regression. We then utilized four matching methods, namely, k-nearest neighbor matching, radius matching, nearest-neighbor matching within the caliper, and kernel matching. Thereafter, the average treatment effect on the treated (ATT) was calculated based on the matched samples. The model is expressed as follows:

(2) $$ATT=E\left(y_{1i}\left|D_{i}=1\right.\right)-E\left(y_{0i}\left|D_{i}=0\right.\right)$$

among them, $D_{i}$ denotes the household fuel choice for the $i_{th}$ individual. To be specific, $D_{i}=1$ represents the usage of polluting fuels, while $D_{i}=0$ indicates their absence. Furthermore, $y_{1i}$ refers to the frailty index of older adults who used polluting fuels and $y_{0i}$ denotes the frailty index of those who did not use such fuels.

Following Frazier [37], the interaction between HPFU and healthy lifestyle behaviors was added to Model 1 to examine the moderating effects of healthy lifestyle behaviors. The modified models are as follows:

(3) $${Frialty}_{i}=\alpha_{1}+\beta_{1}{HPFU}_{i}+\gamma_{1}{Moderator}_{i}+{\eta X}_{i}^{\prime }+\mu_{i}$$

(4) $${Frialty}_{i}=\alpha_{2}+\beta_{2}{HPFU}_{i}+\gamma_{2}{Moderator}_{i}+\delta {HPFU}_{i}\times {Moderator}_{i}+\xi X_{i}^{\prime }+\nu_{i}$$

among them, ${Moderator}_{i}$ represents the moderating variables, which encompass the healthy dietary and social activities; ${HPFU}_{i}\times {Moderator}_{i}$ is created by multiplying HPFU and healthy lifestyle behaviors. The interaction term holds the key to examining moderation and its coefficient stands as the primary focus of our study. If (and only if) this term is significant, we can say that healthy lifestyle behaviors assume the role of a statistically significant moderator of the linear relationship between HPFU and the frailty index [38]. A positive value of δ indicates the positive moderating effects of healthy lifestyle behaviors, while a negative value implies the negative moderating effects of such behaviors.

## 3. Results

### 3.1. Descriptive Statistics

Table 1 presents the sample characteristics. In our study, the maximum value of the frailty index peaked at 0.665, which is consistent with the empirical bounds of the index, typically around 0.7. Among all respondents, 39.3% used polluting fuels for household cooking, predominantly firewood. The respondents had an average age of 82.7 years, whereof 45.8% were male, 50.7% received at least one year of formal education, and 16.3% reported having financial prosperity in comparison to other local people. The last column revealed that the average frailty index for respondents using polluting fuels was higher than those of people using clean fuels (difference among groups=0.010 and p=0.000). Furthermore, significant intergroup differences were noted in terms of ethnicity, marriage, education level, economic status, ADL score, exercise, ventilation, and musty odor (significant at the 10% level of error).

### 3.2. Regression Analysis Results

We employed ordinary least squares (OLS) regression to examine the effects of HPFU on the frailty index among older adults in rural China, as illustrated in Table 2. In the first column, prior to adjusting for control variables, HPFU resulted in an average increase of 0.010 units in the frailty index (p=0.000). Subsequently, individual demographics, socioeconomic status, health risks, and residential environment variables from Table 1 were sequentially included in columns (2) to (5). The results showed a slight change in the coefficients of HPFU on the frailty index but remained statistically significant. By introducing HPFU and all control variables into the final column, we established that, on average, HPFU could elevate the frailty index by 0.006 units (p=0.016). The findings from the regression analysis highlighted the augmented possibility of frailty resulting from the utilization of polluting fuels for household cooking.

### 3.3. Robustness Checks

#### 3.3.1. Propensity Score Matching Results

To minimize the interference of potentially confounding variables in the model and enhance the reliability of the study results, we performed the PSM approach to estimate the ATT effect based on Model 2. Four distinctive matching methods were implemented to determine the homogeneous individuals between those who used polluting fuels and those who used clean fuels for household cooking. As presented in Table 3, HPFU was found to increase the frailty index of older adults by 0.005–0.006 units (significant at the 5% level of error). Additionally, results from the diverse matching methods were basically consistent, thereby validating the robustness of the estimation.

#### 3.3.2. Replacing Estimating Method, Variables, and Samples

Considering that the frailty index is a continuous variable between 0 and 1, we conducted a fracreg logit model for a robustness check. As seen in the first column of Table 4, the results confirmed the statistically significant increase in the frailty index (b=0.037, p=0.017). We then standardized all variables to eliminate the magnitude relationship and set the FI’ as the dependent variable for analysis. Additionally, given the connection between a lower frailty index and better quality of life (QOL), we analyzed the association between HPFU and QOL. As presented in columns (2)–(3), HPFU was associated with both a significant increase in FI’ (b=0.035, p=0.016) and a decrease in QOL (b=−0.126, p=0.000) among older adults. Subsequently, to ensure the representativeness of our findings, we implemented two different methods: removing individuals living alone and randomly sampling 70% of the total sample. The last two columns illustrated the results based on each of these subsamples. Regardless of the method used, the effects of HPFU on the frailty index were significant, with only slight variations in the significance of the coefficients (p=0.023 and p=0.022, respectively). These results affirmed that the findings of our study are robust and stable.

### 3.4. Heterogeneity Analysis

To explore potential variations in the effects of HPFU on the frailty index, we conducted subgroup analyses based on gender, education level, and economic status. As illustrated in Table 5, the OLS results indicated that the effects of HPFU differ across distinct populations. Specifically, our findings revealed a significant association between HPFU and frailty among women (b=0.009, p=0.009) but not among men (b=0.001, p=0.706). Moreover, while HPFU exerted a significant influence on frailty among illiterate participants (b=0.009, p=0.004), no significant relationship was observed among those with higher education (b=0.004, p=0.305). Lastly, our analyses demonstrated a significant association between HPFU and frailty among those with a low-economic status (b=0.007, p=0.006) but not among those with a high-economic status (b=−0.002, p=0.663).

### 3.5. Moderating Effects of Healthy Lifestyle Behaviors

We introduced interaction terms and used Model 3 and Model 4 to investigate the moderating effects of healthy lifestyle behaviors. Considering the potential for the multicollinearity of the interaction terms, we standardized our variables to mitigate any potential statistical errors or confounding factors. Table 6 illustrated the moderating effects of a healthy diet on the relationship between HPFU and the frailty index. The results indicated that a healthy diet had a significant negative effect on the frailty index while controlling for other variables. As detailed in the interaction effect model, our analyses revealed that the coefficients of HPFU×Fresh vegetables, HPFU×Food variety, and HPFU×Balanced diet were −0.035 (p=0.016), −0.032 (p=0.023), and −0.059 (p=0.000), respectively. Despite the nonsignificance of the coefficient for HPFU×Fresh fruits (b=−0.016, p=0.222), we observed a modest, negative moderating effect of daily fresh fruit consumption among older adults.

Similarly, Table 7 displays the moderating effects of social activities on the relationship between HPFU and the frailty index. The results revealed that engaging in typical social activities had a significant negative impact on the frailty index. The coefficients of HPFU×Gardening (b=−0.024, p=0.093) and HPFU×Reading (b=−0.032, p=0.020) were significantly negative, at least at the 10% level of error, while the coefficients of HPFU×Interacting (b=−0.006, p=0.679) and HPFU×Card playing (b=−0.008, p=0.546) were not statistically significant. Taken together, healthy lifestyle behaviors have significant negative moderating effects on the relationship between HPFU and the frailty index. Despite being exposed to HAP from the combustion of polluting fuels, older adults who have healthy dietary habits and participate in social activities may have a lower risk of frailty.

## 4. Discussion

Our study utilized a cross-sectional design to examine the relationship between HPFU and frailty in older adults. The results demonstrated conclusively that HPFU was positively associated with an increased frailty index. Moreover, frailty triggered by the combustion of polluting fuels appeared to be more prevalent among women and individuals with lower levels of education and economic status. Additionally, adopting a healthy diet and participating in social activities could help alleviate the frailty caused by HAP. Against the backdrop of promoting low-carbon transitions in household energy, our findings have significant implications for the prevention and management of frailty in older adults, as well as the promotion of healthy aging.

As a major source of HAP, the association between HPFU and frailty components, such as decreased hand-grip [39] and high rates of obesity [40], have been found to be more prevalent in economically underdeveloped regions. Recent prospective data suggested that the use of coal for heating may increase the frequency of frailty-related hospitalizations by 0.04 per year, resulting in an annual hospital cost of 1195.4 Yuan [41]. Apart from the combustion of polluting fuels, the adverse health effects of HAP due to exposure to secondhand smoke have also been documented [42]. Even for non-smokers, a high serum cotinine level was associated with a higher incidence of pre-frailty and an increased rate of short-term functional decline [43,44]. Several biological mechanisms have been identified as means through which PM impacts human health, including direct or indirect lung inflammation, direct blood translocation, and autonomic regulation [45]. Although the toxicological literature for smoke generated by the combustion of polluting fuels is less extensive than for tobacco smoke, animal studies have reported adverse effects, including cell death and oxidative stress [46,47]. It is speculated that HPFU may initially cause mild disorders of these systems, which over time may lead to the development of frailty. Therefore, reducing and preventing the broader origins of HAP could serve as an effective intervention to protect older adults from the adverse consequences of frailty. The implementation of clean and renewable energy sources, such as solar, wind, and geothermal power, along with the installation of specialized stoves and ventilation equipment, has the potential to alleviate frailty among older adults in developing nations.

HAP is recognized as a “silent killer” for women residing in developing nations. Our heterogeneity analysis revealed that the impact of HPFU on frailty is stronger in females, which further confirms the existence of gender differences in the toxic effects of HAP [48]. Traditional gender roles dictate that women are more likely to assume domestic responsibilities, such as child-rearing and cooking. Conversely, men in rural households exhibited lower 24-h personal exposure to HAP [49]. Importantly, the exposure of infants and young children to HAP is primarily dependent on the caregiver’s involvement in cooking activities. Therefore, reducing the exposure of women in rural areas to HAP can also serve as a means of protecting infants and young children.

In addition, we found that the relationship between HPFU and frailty exhibited a significant marginal effect in illiterate older adults, which confirms the results of previous epidemiological studies [50]. They suggested that HAP exposure may heighten the risk of respiratory illnesses, such as cough and fever, even leading to hospitalization, with those with lower literacy levels being particularly susceptible. One possible explanation might be that individuals who lack formal education tend to have lower health literacy and risk perception. They are less likely to pay attention to the important relationship between household fuel choice and health outcomes. Raufman emphasized that increased awareness of environmental health literacy might help decrease the incidence of HAP-associated symptoms among those who use solid fuels [51]. Accordingly, we propose that education can serve as a viable, long-term strategy to counter the negative consequences of polluting fuel combustion.

Furthermore, as another crucial socioeconomic factor, we found that the effects of HPFU on frailty were significant in older adults with low economic status. Earlier studies supported these findings by indicating that wealth tends to delay the onset and progression of chronic health conditions related to HAP [52]. It is noteworthy that both wealth and education have distinct roles in preventing or mitigating health problems [53]. Education can affect an individual’s intrinsic resources, such as health literacy and risk perception, while wealth can impact the availability of extrinsic health-related resources, such as medication, healthcare, and health management, which are necessary for alleviating frailty. Given that education and economic status remain key determinants of fuel type, it is essential to promote the benefits of clean fuels through multiple channels and implement clean fuel subsidies in rural areas.

Our novel results suggested that adopting healthy lifestyle behaviors may alleviate frailty in older adults who use polluting fuels. This may be because maintaining a healthy lifestyle enhances an individual’s ability to resist risks. Similar studies indicated that in old age, both intellectual activity and social engagement, as well as a healthy diet, may diminish physical vulnerability [54,55] while conferring psychological benefits [56,57]. These can even prevent adverse events, such as hospitalization and mortality, which are considered more serious than frailty [58]. However, certain social activities, such as volunteering, should be moderate rather than frequent [59]. With this in mind, we suggest that incorporating appropriate healthy lifestyle behaviors into future interventions may represent a promising approach to addressing HAP-related frailty. For example, older adults with diabetes should combine nutritional therapy with physical activity, optimal glycemic and metabolic control, and community engagement to maintain their quality of life [60].

We should also note that our study has some limitations. Firstly, our use of cross-sectional data prevented us from conducting a long-term follow-up assessment of frailty status transitions. Despite sampling bias concerns, our findings were consistent with those of previous longitudinal studies [61]. Secondly, all 38 items in our frailty index required in-person responses from older adults. This approach resulted in a considerable number of missing values that could potentially affect parameter estimation. Furthermore, some indicators used employed imprecise measures, which may have led to an under- or over-estimation of the effects. Additionally, important indicators, such as grip strength, gait speed, and serum data reflecting nutrition levels [62], were not available due to data limitations. Furthermore, although we included a variety of healthy dietary indicators and typical social activities, it is necessary to acknowledge that we did not investigate the potential moderating effect of physical activity. Lastly, the CLHLS questionnaire only inquired about the primary cooking fuel type, whereas rural households often use mixed fuel types. Thus, we were unable to classify and study the specificity of fuel combinations in our research. Given the global promotion of clean fuels, further studies could use more accurate observational data with longer follow-ups to investigate whether distinct household fuel consumption patterns or the switch to cleaner fuels can alleviate frailty among older adults.

## 5. Conclusions

Our investigation of older adults in rural China disclosed a correlation between the use of polluting fuels and an increase in frailty, with varied effects observed across different subgroups. Specifically, the impact of polluting fuels proved to be more pervasive among women, individuals with a lower educational attainment, and those with an inferior economic status. Conversely, men, individuals with a higher educational attainment, and those with a superior economic status exhibited a comparatively lesser impact. Moreover, our findings underscored the significant role of healthy lifestyle behaviors in alleviating the effects of this association. Considering the social outcomes and medical burden of frailty, researchers and stakeholders should direct their targeted attention to older adults, particularly women, those with a low educational attainment, and those with a low socioeconomic status. Intervention measures should also take healthy lifestyle behaviors into consideration to alleviate HAP-related frailty in older adults who use polluting fuels.

## Figures and Tables

**Table 1:** Descriptive statistics of the investigated variables.

Variable	Full Sample (N = 4535)	Treated Group (N = 1780)	Control Group (N = 2755)	*t*-test
Frailty index	0.179 (0.078)	0.185 (0.080)	0.175 (0.076)	0.010 ***
Gender	0.458 (0.498)	0.444 (0.497)	0.466 (0.499)	−0.023
Age	82.708 (11.418)	82.738 (11.320)	82.689 (11.484)	0.049
Ethnicity	0.927 (0.261)	0.915 (0.279)	0.934 (0.248)	−0.019 **
Marriage	0.483 (0.500)	0.520 (0.500)	0.460 (0.498)	0.061 ***
Illiteracy	0.507 (0.500)	0.554 (0.497)	0.476 (0.500)	0.079 ***
Primary education	0.379 (0.485)	0.362 (0.481)	0.391 (0.488)	−0.029 *
Middle school education and above	0.114 (0.318)	0.084 (0.277)	0.134 (0.340)	−0.050 ***
Financial independent	0.356 (0.479)	0.354 (0.478)	0.356 (0.479)	−0.002
Economic status	0.163 (0.369)	0.112 (0.316)	0.196 (0.397)	−0.083 ***
ADL score	11.510 (1.542)	11.557 (1.496)	11.479 (1.570)	0.078 *
Smoking	0.185 (0.388)	0.183 (0.387)	0.185 (0.389)	−0.002
Drinking	0.166 (0.372)	0.170 (0.375)	0.164 (0.370)	0.006
Exercise	0.293 (0.455)	0.242 (0.428)	0.326 (0.469)	−0.083 ***
Ventilation	0.894 (0.308)	0.863 (0.344)	0.914 (0.281)	−0.051 ***
Musty odor	0.151 (0.358)	0.197 (0.398)	0.122 (0.327)	0.075 ***

Note: *** *p* < 0.01; ** *p* < 0.05; * *p* < 0.1; Standard errors are reported in parentheses.

**Table 2:** The effects of household polluting fuel use on the frailty index.

Variable	Dependent Variable: Frailty Index				
	(1)	(2)	(3)	(4)	(5)
HPFU	0.010 *** (0.002)	0.010 *** (0.002)	0.007 *** (0.002)	0.008 *** (0.002)	0.006 ** (0.002)
Individual demographics	No	YES	YES	YES	YES
Socioeconomic status	No	No	YES	YES	YES
Health risks	No	No	No	YES	YES
Residential environment	No	No	No	No	YES
R-square	0.0039	0.047	0.067	0.106	0.120
Observation	4535	4535	4535	4535	4535

Note: *** *p*< 0.01; ** *p* < 0.05; robust standard errors are provided within parentheses; “YES” indicated that the variables were included in the model and “No” indicated that they were not included.

**Table 3:** PSM estimation results.

Variables	Dependent Variable: Frailty Index			
	k-Nearest Neighbor Matching	Radius Matching	Nearest-Neighbor Matching within Caliper	Kernel Matching
	(1)	(2)	(3)	(4)
HPFU	0.005 ** (0.003)	0.006 ** (0.002)	0.006 ** (0.003)	0.005 ** (0.002)
Individual demographics	YES	YES	YES	YES
Socioeconomic status	YES	YES	YES	YES
Health risks	YES	YES	YES	YES
Residential environment	YES	YES	YES	YES
Observation	4535	4535	4535	4535

Note: ** *p* < 0.05; Standard errors are reported in parentheses; “YES” indicated that the variables were included in the model.

**Table 4:** Results of robustness check.

Variables	Replacing Estimating Method	Replacing Dependent Variables		New-Generated Subsamples	
	(1)	(2)	(3)	(4)	(5)
HPFU	0.037 ** (0.016)	0.035 ** (0.014)	−0.126 *** (0.023)	0.006 ** (0.003)	0.006 ** (0.003)
Individual demographics	YES	YES	YES	YES	YES
Socioeconomic status	YES	YES	YES	YES	YES
Health risks	YES	YES	YES	YES	YES
Residential environment	YES	YES	YES	YES	YES
Observation	4535	4535	4532	3737	3175

Note: *** *p* < 0.01; ** *p* < 0.05; Standard errors are reported in parentheses; “YES” indicated that the variables were included in the model.

**Table 5:** Heterogeneity analysis of household polluting fuel use on frailty index.

Variables	Gender		Education Level		Economic Status	
	Women	Men	Illiterate	Educated	Low	High
	(1)	(2)	(3)	(4)	(5)	(6)
HPFU	0.009 *** (0.003)	0.001 (0.003)	0.009 *** (0.003)	0.004 (0.004)	0.007 *** (0.003)	−0.002 (0.005)
Individual demographics	YES	YES	YES	YES	YES	YES
Socioeconomic status	YES	YES	YES	YES	YES	YES
Health risks	YES	YES	YES	YES	YES	YES
Residential environment	YES	YES	YES	YES	YES	YES
Observation	2460	2075	2298	2237	3796	739

Note: *** *p* < 0.01; Standard errors are reported in parentheses; “YES” indicated that the variables were included in the model.

**Table 6:** The moderating effects of a healthy diet.

Variables	Dependent Variable: Frailty Index							
	Fresh Vegetables		Fresh Fruits		Food Variety		Balanced Diet	
	(1)	(2)	(3)	(4)	(5)	(6)	(7)	(8)
HPFU	0.031 ** (0.014)	0.030 ** (0.014)	0.033 ** (0.014)	0.032 ** (0.014)	0.028 * (0.015)	0.026 * (0.015)	0.024 * (0.014)	0.020 (0.014)
Healthy dietary	−0.079 *** (0.015)	−0.078 *** (0.015)	−0.077 *** (0.013)	−0.079 *** (0.013)	−0.097 *** (0.014)	−0.099 *** (0.014)	−0.117 *** (0.014)	−0.121 *** (0.014)
HPFU × Healthy dietary		−0.035 ** (0.014)		−0.016 (0.013)		−0.032 ** (0.014)		−0.059 *** (0.014)
Individual demographics	YES	YES	YES	YES	YES	YES	YES	YES
Socioeconomic status	YES	YES	YES	YES	YES	YES	YES	YES
Health risks	YES	YES	YES	YES	YES	YES	YES	YES
Residential environment	YES	YES	YES	YES	YES	YES	YES	YES
Observation	4530	4530	4530	4530	4448	4448	4496	4496

Note: *** *p* < 0.01; ** *p* < 0.05; * *p* < 0.1; Standard errors are reported in parentheses; Regression model with standardized variables; “YES” indicated that the variables were included in the model.

**Table 7:** The moderating effects of social activities.

Variables	Dependent Variable: Frailty Index							
	Gardening		Reading		Interacting		Card-Playing	
	(1)	(2)	(3)	(4)	(5)	(6)	(7)	(8)
HPFU	0.033 ** (0.014)	0.033 ** (0.014)	0.033 ** (0.014)	0.031 ** (0.014)	0.036 ** (0.014)	0.036 ** (0.014)	0.034 ** (0.014)	0.034 ** (0.014)
Social activities	−0.031 ** (0.014)	−0.034 ** (0.014)	−0.038 *** (0.014)	−0.044 *** (0.015)	−0.029 * (0.015)	−0.029 * (0.015)	−0.063 *** (0.014)	−0.063 *** (0.014)
HPFU × Social activities		−0.024 * (0.014)		−0.032 ** (0.014)		−0.006 (0.014)		−0.008 (0.013)
Individual demographics	YES	YES	YES	YES	YES	YES	YES	YES
Socioeconomic status	YES	YES	YES	YES	YES	YES	YES	YES
Health risks	YES	YES	YES	YES	YES	YES	YES	YES
Residential environment	YES	YES	YES	YES	YES	YES	YES	YES
Observation	4532	4532	4530	4530	4531	4531	4533	4533

Note: *** *p* < 0.01; ** *p* < 0.05; * *p* < 0.1; Standard errors are reported in parentheses; Regression model with standardized variables; “YES” indicated that the variables were included in the model.

## Appendix A

**Table A1:** List of 38 items included in the frailty index.

No.	Items	Coding of Variables
1	Self-rated health	Very poor = 1.00; poor = 0.75; fair = 0.50; good = 0.25; excellent = 0.00
2	Health change in the past year	Much worse = 1.00; slightly worse = 0.75; almost the same = 0.50; slightly better = 0.25; much better = 0.00
3	Interviewee-rated health	Very ill = 1.00; moderately ill = 0.67; relatively healthy = 0.33; surprisingly healthy = 0.00
4	Hearing function: in which ear(s) do you have a hearing difficulty?	Both = 1.00; right = 0.50; left = 0.50; neither = 0.00
5	Visual function: can you see the break in the circle?	Blind = 1.00; cannot see = 0.67; can see but not distinguish = 0.33; can see and distinguish = 0.00
6	Sleep quality	Very poor = 1.00; poor = 0.75; fair = 0.50; good = 0.25; excellent = 0.00
7	Brush teeth rarely or never	Yes = 1.00; no = 0.00
8	Heart rhythm, beats per min	<60 or>100 = 1.00; ≥60 and ≤100 = 0.00
9	Waist circumference to hip circumference ratio	≥0.95 for men or ≥0.90 for women = 1.00; ≥0.90 and <0.95 for men or ≥0.85 and <0.90 for women = 0.50; <0.90 for men or <0.85 for women = 0.00
10	Body-mass index (kg/m^2^)	<18.5 or ≥28.0 = 1.00; ≥24.0 and <28.0 = 0.50; ≥18.5 and <24.0 = 0.00
11	Number of serious illnesses in the past 2 years	≥2 times = 2.00; 1 time = 1.00; 0 time = 0.00
12	Suffering from hypertension	Yes = 1.00; no = 0.00
13	Suffering from diabetes	
14	Suffering from tuberculosis	
15	Suffering from heart disease	
16	Suffering from stroke/cerebrovascular disease	
17	Suffering from bronchitis, emphysema, asthma, or pneumonia	
18	Suffering from cancer	
19	Suffering from arthritis	
20	Suffering from bedsores	
21	Suffering from gastric or duodenal ulcers	
22	Suffering from Parkinson’s disease	
23	Suffering from cholecystitis or cholelith	
24	Suffering from chronic nephritis	
25	Feeling worried about some small things	Always = 1.00; often = 0.75; sometimes = 0.50; seldom = 0.25; never = 0.00
26	Difficult to concentrate	
27	Feeling sad or depressed	
28	Feeling useless with age	
29	Feeling nervous and scared	
30	Feeling lonely	
31	Feeling unable to continue life	
32	Feeling uneasy, worried and annoyed	Almost every day = 1.00; more than half of days = 0.67; for several days = 0.33; never = 0.00
33	Can’t stop or can’t control worry	
34	Worrying too much about all kinds of things	
35	Being very nervous and it is difficult to relax	
36	Being very anxious and can’t sit still	
37	Becoming easy to get annoyed or easily irritated	
38	Feeling like something terrible happens	

Notes: Participation reporting two or more serious illnesses was assigned a value of 2.00; Body-mass index was calculated by dividing the weight (kg) of an individual by their height (m^2^).
